# It’s not all about COVID‐19: pneumocystis pneumonia in the era of a respiratory outbreak

**DOI:** 10.1002/jia2.25533

**Published:** 2020-06-01

**Authors:** Chiaw Yee Choy, Chen Seong Wong

**Affiliations:** ^1^ National Centre for Infectious Diseases (NCID) Singapore Singapore; ^2^ Department of Infectious Diseases Tan Tock Seng Hospital Singapore Singapore; ^3^ Yong Loo Lin School of Medicine National University of Singapore Singapore Singapore

**Keywords:** PCP, COVID‐19, outbreak, opportunistic infections, HIV, diagnosis

Dear Editors:

As the COVID‐19 pandemic continues to spread [1], hospitals have turned their focus to diagnosing COVID‐19 in patients who present with respiratory symptoms, and there is a risk of missing other differential diagnoses. We present two cases of newly diagnosed advanced HIV infection with *Pneumocystis* pneumonia (PCP) that were initially managed as suspect cases of COVID‐19, and in whom HIV was not initially considered.

The first case is of a 62‐year‐old male with a history of stage III undifferentiated nasopharyngeal carcinoma and hypothyroidism. He reported no recent travel outside of Singapore in the previous month and no contact with confirmed COVID‐19 cases. He presented with complaints of shortness of breath and diarrhoea for two weeks with generalized lethargy for one month. On admission, he required supplemental oxygen via nasal cannula at 2 L per minute, but his vital signs were otherwise stable.

The second case is of a 20‐year‐old female who recently returned from the United Kingdom (UK). She has a history of childhood asthma for which she was not on treatment. She reported cough of three months duration prior to admission with intermittent fever and shortness of breath. However, she developed persistent fever and worsening breathlessness one week prior to admission. Her vital signs were stable on admission.

Both cases were admitted and managed with the provisional diagnosis of COVID‐19 infection, and followed very similar courses throughout their stay. Both had lymphopenia and raised lactate dehydrogenase (LDH) levels with radiological features suggestive of COVID‐19 pneumonia. In view of this, both patients had three sets of SARS‐CoV‐2 real‐time polymerase chain reaction performed on nasopharyngeal swab samples 24 hours apart, all of which returned negative. Both patients subsequently deteriorated on the third inpatient day, requiring increasing supplemental oxygen.

A computed tomography (CT) of the chest for the first case showed widespread patchy ground glass changes seen in both lungs with upper lobe predominance and peripheral consolidation in the right lower lobe. The CT chest scan of the second case showed extensive ground glass opacities in both lungs with multi‐lobar involvement, worst in the perihilar regions, sparring the subpleural regions and the lung bases.

On focused re‐examination, both cases were found to have oral thrush. An HIV antigen‐antibody screening test was then performed for both patients, and both returned positive. On the basis of their clinical and radiologic findings, both patients were started on trimethoprim‐sulfamethoxazole (TMP‐SMX) with prednisolone for severe presumptive PCP. Both patients improved dramatically and were subsequently discharged after 16 days for case 1 and 18 days for case 2 with plans for outpatient follow up for HIV care.

PCP and COVID‐19 share numerous characteristics. Both diseases present with fever, fatigue, dry cough and dyspnoea [2, 3]. In terms of chest CT findings, bilateral symmetrical ground glass opacities are frequently seen on chest CT scans in both PCP and COVID‐19 [4, 5, 6]. PCP frequently occurs when the CD4 count drops below 200 cells/μL, which can manifest as lymphopenia [7]. Leucopoenia also appears to be the most common presentation in patients with COVID‐19, although leucocytosis and lymphopenia have also been reported [8, 9]. Elevated LDH has also been reported in both COVID‐19 and PCP [7, 10, 11]. In both cases, the presence of elevated LDH is an indicator of poorer prognosis [7, 11, 12].

However, PCP generally follows a more subacute course than COVID‐19, particularly when considering milder forms of the disease, with 33% of patients having symptoms for at least two to four weeks prior to presentation in a case series from early in the HIV epidemic [3]. In comparison, the median time to development of dyspnoea or pneumonia for symptomatic COVID‐19 has been reported to be eight to nine days from illness onset [2, 8]. Dyspnoea is also a more prominent symptom in PCP and was seen in up to 95% of patients in one case series while it is only seen in about 11% to 31% of patients with COVID‐19 [2, 3, 9, 13]. Oral thrush, which has a strong association with PCP, has not been seen in COVID‐19 [14]. In terms of radiological findings, a systematic review of 919 patients with COVID‐19 found that the most characteristic manifestations seen were ground glass opacification frequently seen in the peripheries of the lungs (76.0%), with a predilection for multi‐lobar distribution (78.8%) [15]. In contrast, the PCP‐related ground glass opacities seen on chest CT tend to spare the lung peripheries and may be associated with cyst formation and spontaneous pneumothorax [16, 17].

Although the similarities between the two diseases might explain why the diagnosis of PCP was initially missed, there are also other reasons for the delay in HIV diagnosis in these two patients. Firstly, PCP with HIV infection is less commonly seen in settings with access to antiretroviral therapy (ART), with the majority of cases previously occurring in young children within the first year of life and male adults above the age of 50 years in the pre‐ART era [18, 19]. Case 2, who is a 20‐year‐old female with a significant travel history to the UK, did not fit the typical demographic for PCP. This highlights the importance of considering HIV as a diagnosis, and offering HIV testing to all adults as part of universal screening, as well as part of directed screening in patients presenting with features suggestive of indicator conditions, including opportunistic infections. Moreover the patients’ histories of nasopharyngeal cancer and asthma, respectively, may also have played a role in HIV not initially being considered as a possible presenting diagnosis. Secondly, the most common differential diagnosis of ground glass changes in CT chest is viral pneumonitis, such as that caused by adenovirus and influenza, which may explain why the diagnosis of PCP was initially not considered [20, 21]. However, while the incidence of HIV has been declining in Singapore, 50% of the newly diagnosed cases had late stage HIV infection upon diagnosis, putting them at higher risk of conditions like PCP. [22]. In addition, the rates of PCP in Singapore have not significantly decreased despite the advent of ART, highlighting the importance of remaining vigilant for cases of opportunistic infection [23]. Finally, as the diagnosis of PCP and underlying immunosuppression was not initially considered, the presence of oral thrush was also missed in both cases.

It is understandable that the first differential for a patient with respiratory symptoms is COVID‐19 during this outbreak. However, there are other respiratory pathogens which may mimic the presentation of COVID‐19. As illustrated in this letter, PCP shares numerous characteristics with COVID‐19, although there are some key differences. We hope to highlight the importance of considering this diagnosis in individuals presenting with suggestive symptoms, so that cases of potential HIV infection are not missed during this outbreak.

## Competing interests

All authors have no conflict of interest to declare.

## Authors’ contributions

CYC and CSW contributed to the design of the manuscript. CYC drafted the manuscript with input from CSW. All authors have read and approved the final manuscript.
